# Orbital mucormycosis in an immunocompetent individual

**Published:** 2012-12

**Authors:** P Badiee, Z Jafarpour, A Alborzi, P Haddadi, M Rasuli, M Kalani

**Affiliations:** 1 Prof. Alborzi Clinical Microbiology Research Center; 2 Student Research Committee, Shiraz University of Medical Sciences, Shiraz, Iran

**Keywords:** mucormycosis, immunocompetent, orbital exentration, amphotericin B

## Abstract

**Background:**

Orbital mucormycosis caused by Zygomycetes is a rare and fatal infection that generally affects the patients who are immunocompromised. Despite antifungal therapy and aggressive surgical intervention, mucormycosis can cause serious and rapidly fatal infections if delayed diagnosis or therapeutic management occurs. Here, we report orbital mucormycosis in a healthy boy, with a favorable outcome after aggressive treatment. He has had no recurrence since the end of his treatment.

**Case present:**

A 2-year old healthy boy, some days after entry of dust particle to his left eye presented with swelling and redness of the eye. With diagnosis of “periorbital cellulitis” intravenous antibiotics vancomycin (40 mg/kg/day) and ceftriaxone (75 mg/kg/day) were started but no improvement was observed. The results of biopsy and tissue culture led us to a diagnosis of mucormycosis. Orbital exenteration, combined with intravenous amphotericin B (1 mg/kg/day), resulted in the patient's survival.

**Conclusion:**

Due to the high mortality rate of mucormycosis, early diagnosis based on clinical findings and biopsy could be effective for management of the patients suffering from this infection.

## INTRODUCTION

Mucormycosis is a life threatening and opportunistic fungal infection caused by fungi of the *Mucorales* order belonging to the *Phycomycete*s classof which, the main pathogens belong to the *Rhizomucor, Rhizopus, Absidia* and *Mucor* species (1, 2). This infection is uncommon but the incidence appears to be increasing (1, 3, 4). Dounia Bitar et al. documented an increasing incidence of zygomycosis from 0.7/million in 1997 to 1.2/million in 2006 (p < 0.001) (5). Despite antifungal therapy and aggressive surgical intervention, mucormycosis can cause serious and rapidly fatal infections if delayed diagnosis or therapeutic management occurs. Here, we report orbital mucormycosis in a healthy boy, with a favorable outcome after aggressive treatment.

## CASE REPORT

**Fig. 1:**
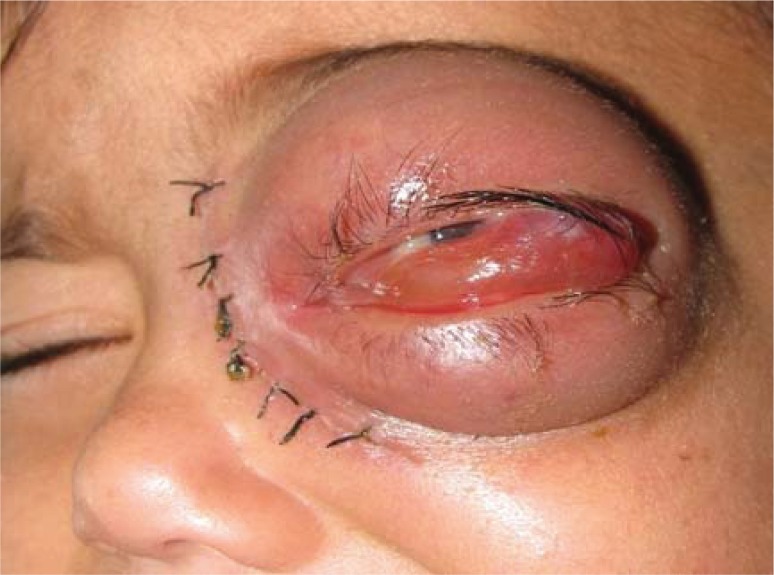
Swelling of upper and lower lid in the patient with mucormycosis.

A 2-year old boy was referred to Nemazee hospital, Shiraz University of Medical Sciences, Shiraz, Iran in the spring 2010 with 18 days history of swelling and redness of left eye. He had history of the entry of some dust particle into his left eye, after that a redness of left eye developed that aggravated during a week. He had been admitted to another hospital and periorbital cellulitis was diagnosed and the patient received vancomycin (40 mg/kg/day) and ceftriaxone (75 mg/kg/day) for 10 days, but no improvement was observed and referred to Nemazee hospital. At examination, swelling of upper and lower lids, more prominent on lower part was seen (Fig. 1). Proptosis, conjunctival congestion and chemosis and limitation of motion in all directions were seen. Clindamycin (40 mg/kg/day) and ceftriaxone (75 mg/kg/day), was initiated. For measurement of visual acuity and goinoscopy, he was not cooperative. Pupil shape, reactivity, and size and lacrimal drainage system were normal, and nystagmus was negative. His past medical history was significant only for chronic itching of eyes prior first admission.

Laboratory investigation revealed: white cell count 18.2×10^3^/mm^3^, neutrophil:32%, lymphocyte 50%, monocyte 14%, eosinophil 4%, hemoglobin 10.4 g/dL, mean corpuscular volume (MCV) 67.1fl, mean corpuscular hemoglobin (MCH) 18.6 pg, platelet count 152000/mm^3^, ESR 25 mm/1^st^hour, C. reactive protein 6, blood sugar 85 mg/dl. Total cerebrospinal fluid (CSF) cell count was 10 including 2 white blood cell (all lymphocyte). The sugar and protein of CSF were 69 and 22 mg/dl, respectively. Gram staining of CSF did not show any organism. Other rare disorders that present with proptosis such as Wegener granulomatosis, leukemia, Burkitt lymphoma and histiocytosis were investigated. Peripheral antineutrophil cytoplasmic antibodies (P-ANCA) and C-Anti-neutrophil cytoplasmic antibodies (C-ANCA) were negative and bone marrow aspiration and trephine biopsy revealed normocellular marrow with no evidence of metastasis. Immunohistochemical study for S_100_, CD_30_, CD_20_, CD_3_ and BCL-2, myeloperoxidase was negative in histocyte-like cells.

**Fig. 2:**
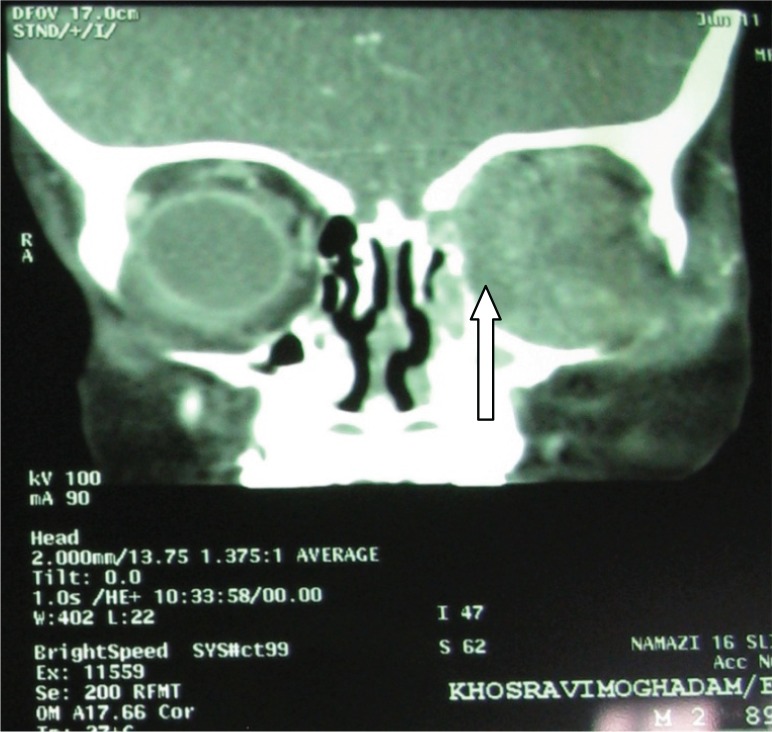
Computer tomography scan of the orbits of patient with mucormycosis. It demonstrated partly cystic solid occupying lesion with central necrosis in the medial aspect of left orbit with irregularity of the wall.

Computer tomography scan of the orbits demonstrated partly cystic solid occupying lesion in the medial aspect of left orbit with compression of the optic nerve and irregularity of the wall with central necrosis detected (Fig. 2).

**Fig. 3:**
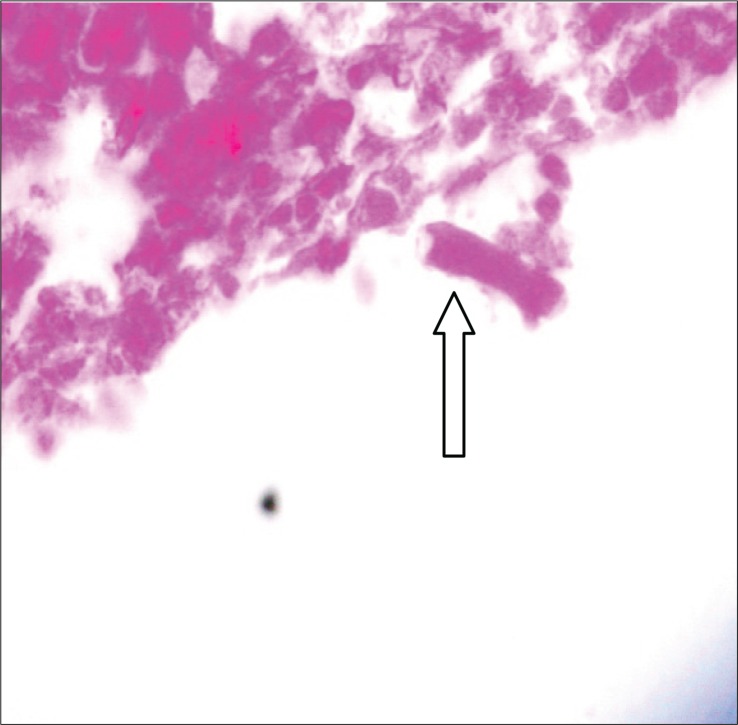
Orbital tissue showing typical non septate hyphae.

**Fig. 4:**
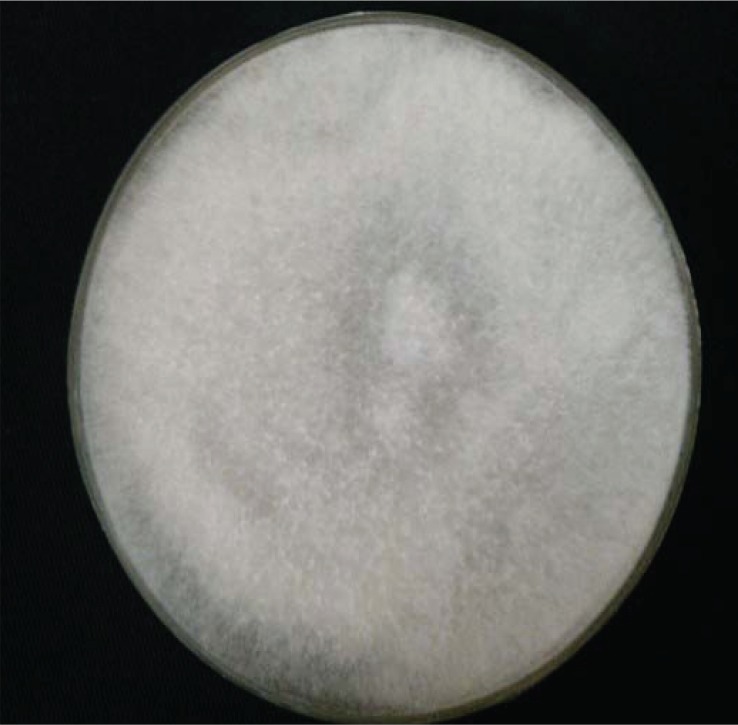
*Mucor* spp. isolated from tissue culture.

**Fig. 5:**
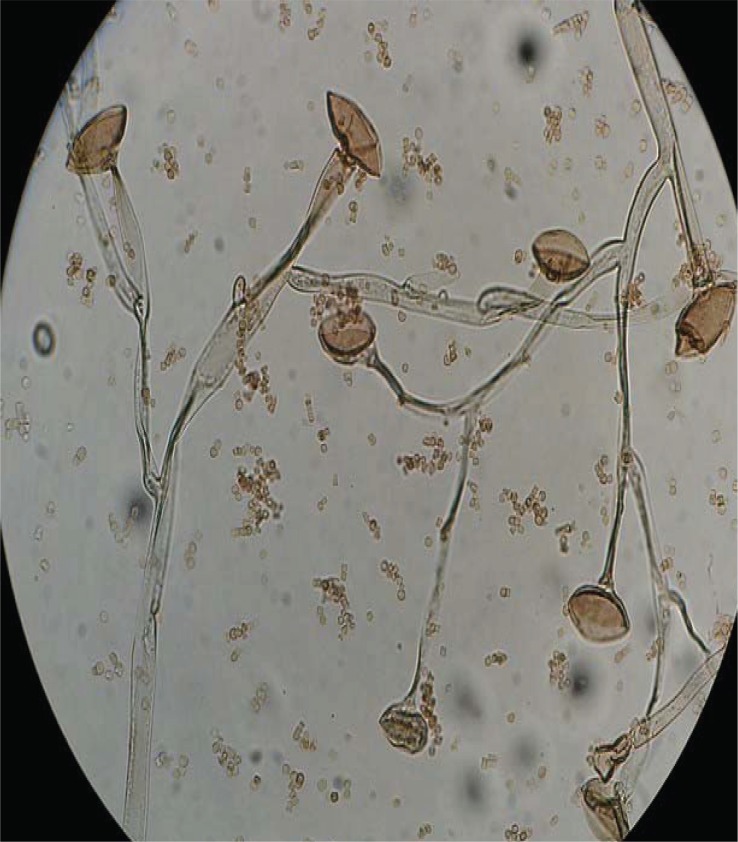
*Mucor* species-microscopic morphology, 400x Lactophenol cotton blue.

Anterior orbitotomy and orbital mass biopsy of the left eye were done, no evidence of pus was detected and only necrotic tissue was seen. Four pieces of creamy-white, rubbery tissue was removed and sent to pathology and mycology labs. The pathology result was reported as vague aggregates of histiocytes with perivascular lymphocyte. Some areas of necrosis with hyphen elements were detected. In the mycology lab, direct smear of tissue with potassium hydroxide showed non-septated hyphae (Fig. 3) and after culture the sample on Sabouraud Dextrose Agar (Merck, Darmstadt, Germany); diagnosis was documented with growth of Mucor in culture (Figs. 4, 5). Immunological studies revealed normal levels of IgG_1_, IgG_2_, IgG_3_, IgG_4_, IgA, C_3_, C_4_, CD^+^_3_ ( T-cell), CD^+^_4_, (helper T-cell), CD^+^_8_(cytotoxic T-cell), CD^+^16, CD^+^_4_/ CD^+^_8_ ratio, B-cell, and Natural killer cell. HIV test, blood and sinus culture results were negative.

Surgical exenteration of the left eye was done and amphotericin B (1 mg/kg) and clindamycin (30 mg/kg) daily were started for him. He was discharged after about 4 weeks and treatment was extended with itraconazole (5 mg/kg/day) for 6 months. Two-year follow up examination revealed no evidence of recurrence.

### Ethical Consideration

The ethics committee of Clinical Microbiology Research Center, Shiraz University of Medical Sciences has reviewed and approved the study regarding the patient written consents before participating in the study.

## DISCUSSION

Mucormycosis caused by Zygomycetes is a rare and fatal infection that generally affects the patients who are immunocompromised. It generally affects almost exclusively the patients with known predisposing conditions, such as poorly controlled diabetes, long term consumption of steroid, antibiotics, and cytotoxic agents, leukemia, lymphoma, organ transplantation, severe burns, hemochromatosis and use of deferoxamine and possibly malnutrition considering the majority of cases occurring in the developing nations (6, 7). Furthermore, previous use of antifungal drugs lacking activity against *Zygomycetes* has coincided with an increase in the incidence of *Mucorals* (5, 8). In the present study, the evaluation of immunological factors revealed that the patient was healthy with non-risk factor for mucormycosis. We also found in the literature some cases of primary fungal infections (9) and mucormycosis in the patients with no underlying disease; in the breast (10), maxillary first molar (11) gastrointestinal (12), and in rhino-orbital-cerebral mucormycosis after a high pressure water jet injury (13). The interior of the eye does not contain lymphoid vessels but is highly vascularized, so that in healthy persons ocular immune system is sufficient to prevent infection except cases of massive contamination with traumatic inoculation (8, 14). The patient in the present study had only the history of the entry of dust particle into his eye and itching led it to deeper layers of the eye.

Although there have been reports of mucormycosis, true orbital infection in healthy individuals is very rare (13–16).

Although molecular and serological methods for the diagnosis of some etiologic agents causing invasive fungal infections have been reported (17–19), the gold standard for the diagnosis of mucormycosis like other fungal diseases is tissue culture and in this study, the causative agent was isolated from the culture media. Unfortunately, no serological method is available for the diagnosis of mucormycosis, and there are very few reports on the molecular method for the diagnosis of this infection (20).

Chamilos G et al. showed that early diagnosis of mucormycosis and initiation of appropriate therapy within 5 days contributed to the improvement in the respective patient's survival, compared with the initiation of therapy at ≥6 days (83% vs. 49% survival) (21). Standard treatment in immunocompromised patients requires discontinuation of immunosuppressive drugs like cyclosporine or mycophenolatemofetil, along with aggressive surgical debridement and systemic antifungal therapy. Despite advances in diagnosis and aggressive surgical and polyene antifungal therapy, a high mortality rate of 30-70% still exists for the disease (22). The modalities of treatment are rapid diagnosis, surgical intervention, and antifungal agents (23, 24). Due to some areas of necrosis and poor penetration of amphotericin B into the blood brain barrier, surgical debridement was necessary (25). Lipid formulations of amphotericin B (LFABs) are less nephrotoxic and can be used as antifungal agent at higher doses and a longer time than amphotericin B (26, 27). In this study, as the patient had the normal immunological condition, after exenteration of left eye, amphotericin B (1 mg/kg) was started in the hospital and then itraconazole was extended for 6 months. There is a report on a successful therapy for a healthy patient, treated with debridement and fluconazole (11). Due to the high mortality rate of mucormycosis, early diagnosis based on clinical findings and biopsy could be very effective for management of the patients suffering from this infection.
